# Supplementary material for: The MS Remyelinating Drug Bexarotene (an RXR Agonist) Promotes Induction of Human Tregs and Suppresses Th17 Differentiation In Vitro
Source: Front Immunol. 2021 Aug 10;12:712241. doi: 10.3389/fimmu.2021.712241 (PMC8382874; doi:10.3389/fimmu.2021.712241)
Supplement: Supplementary file 1 [file DataSheet_1.pdf]

Supplemental data

A

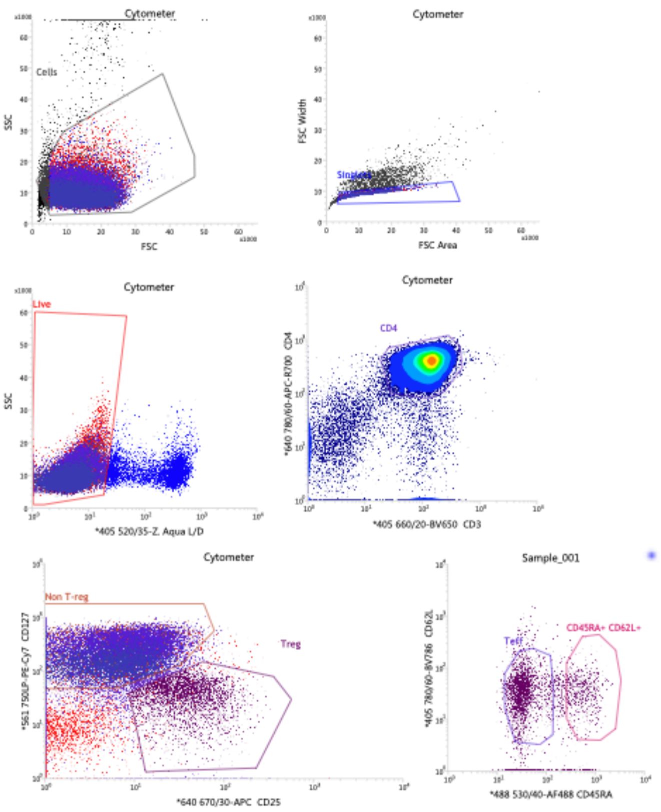

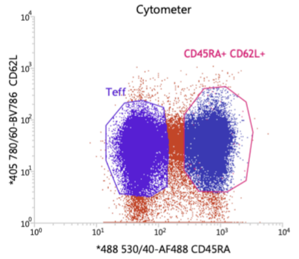

Populations: Cytometer

Populations	Even...	% Total	% Parent
All Events	100,000	100.00%	####
Cells	98,436	98.44%	98.44%
Singlets	89,325	89.32%	90.74%
Live	79,227	79.23%	88.70%
CD4	71,072	71.07%	89.71%
Non T-reg	63,627	63.63%	89.52%
CD45RA+...	20,034	20.03%	31.49%
Naive DP	19,562	19.56%	97.64%
Teff	28,509	28.51%	44.81%
Treg	4,390	4.39%	6.18%

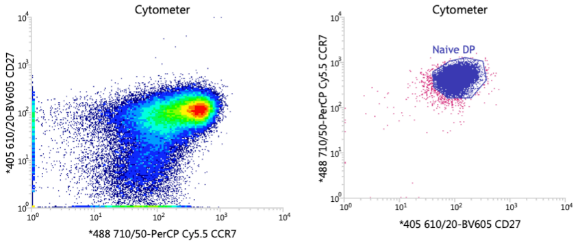

B

Sorting Panel

530/30	CD45RA	AF488
695/40	CCR7	PerCP-Cy5.5
525/50	Zombie Aqua	BV510
605/12	CD27	BV605
650/8	CD3	BV650
780/60	CD62L	BV786
585/15	FOXP3	PE
780/60	CD127	PE-Cy7
670/14	CD25	APC
730/45	CD4	APC-R700

1. nTregs
(CD3+ CD4+ CD25hi CD127-)

2. Teffs
(CD3+ CD4+ CD127+ CD45RA-)

3. CD4+ Naive T cells
(CD3+ CD4+ CD127+ CD45RA+ CD62L+ CCR7+ CD27+)

S1. (A) Naïve CD4+ lymphocyte sorting report and (B) corresponding sorting antibody panel.

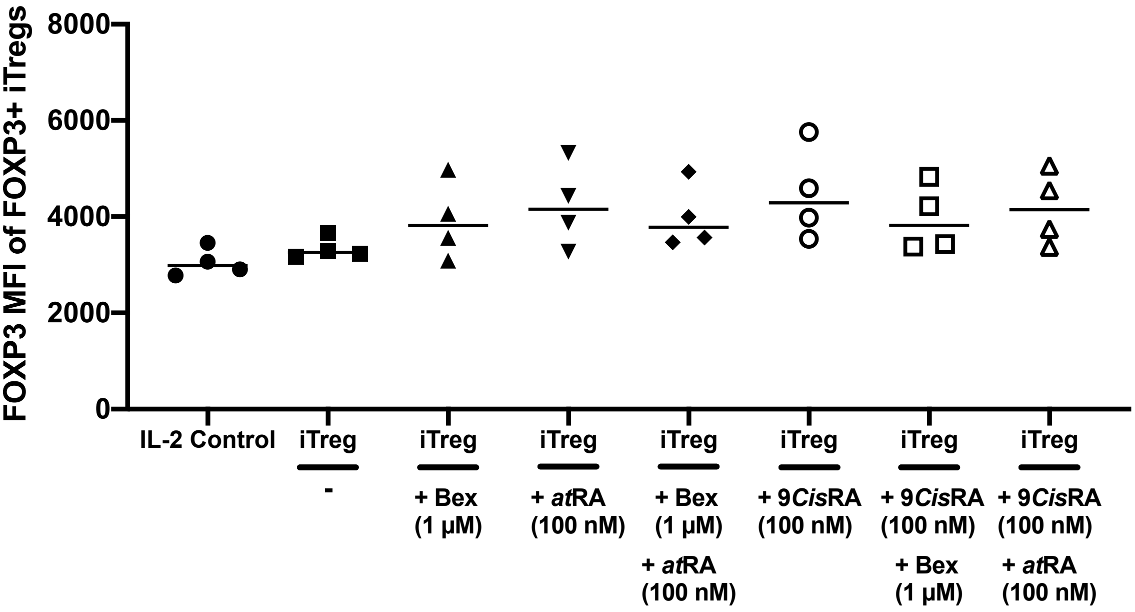

S2. Quantified MFI of FOXP3+ iTregs under different treatment conditions.

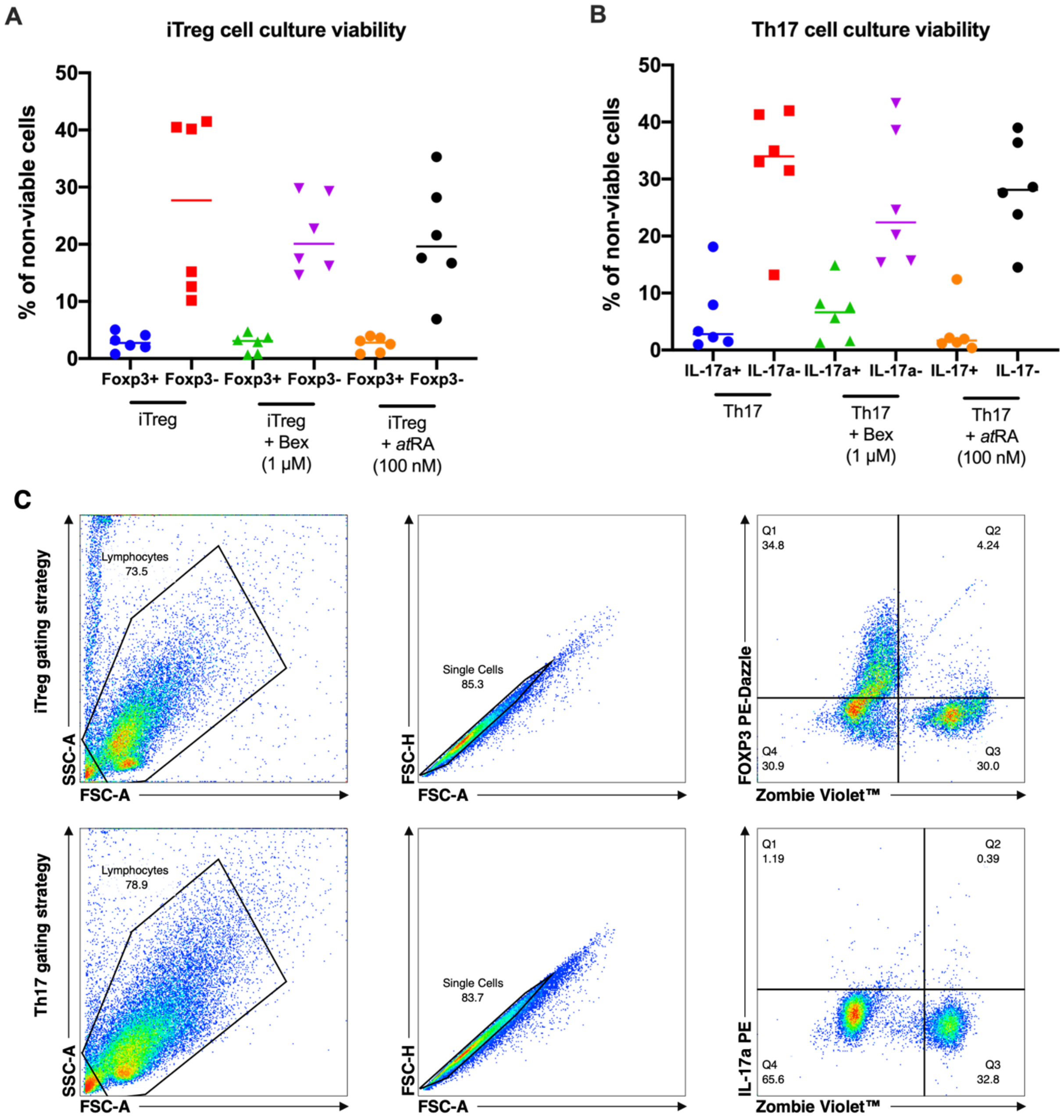

S3. (A) % of non-viable cells that are Foxp3$^{+/-}$ from different iTreg culture conditions (± Bexarotene 1µM, *at*RA 100nM). (B) % of non-viable cells that are IL17a$^{+/-}$ from different Th17 culture conditions (± Bexarotene 1µM, *at*RA 100nM). (C) representative gating strategies for viability data.

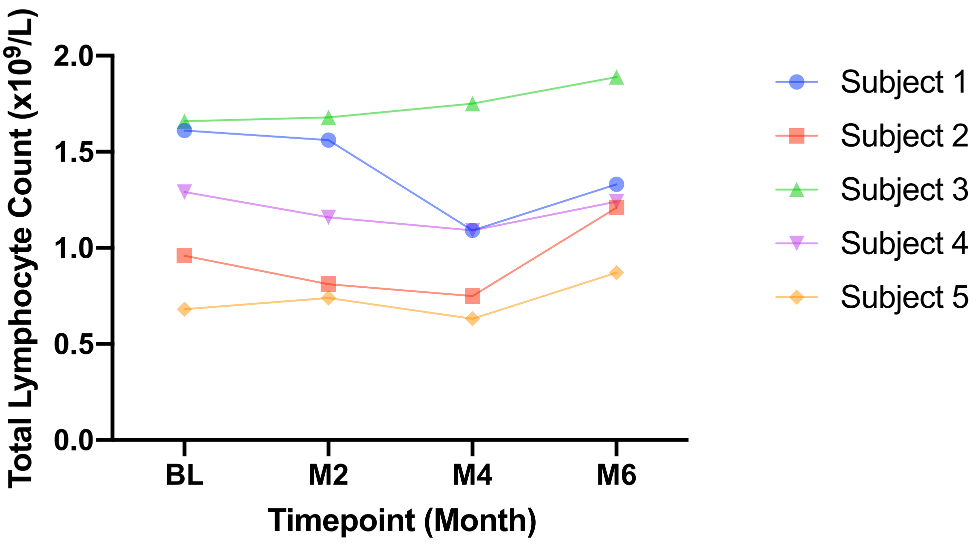

S4. Total lymphocyte count from subjects enrolled within the CCMR-One trial used in analysis.
