# Supplementary material for: The MS Remyelinating Drug Bexarotene (an RXR Agonist) Promotes Induction of Human Tregs and Suppresses Th17 Differentiation In Vitro
Source: Front Immunol. 2021 Aug 10;12:712241. doi: 10.3389/fimmu.2021.712241 (PMC8382874; doi:10.3389/fimmu.2021.712241)
Supplement: Supplementary file 2 [file DataSheet_2.pdf]

Table 1. Baseline Characteristics

	**Bexarotene**	**Placebo**
Total number of participants<br>Cambridge, number (%)<br>Edinburgh, number (%)	25<br>16 (64)<br>9 (36)	24<br>15 (63)<br>9 (37)
Age, years; mean (SD)	40·4 (6·2)	38 (6·8)
Sex<br>Female<br>Male	 15<br>10	 13<br>11
Disease duration, years; mean (SD)	11 (5·9)	8·4 (5·8)
Number of relapses in last 2 years; median (quartiles)	0 (0, 1)	0·5 (0, 1·3)
EDSS; median (quartiles)	2·5 (1·5, 3·5)	2·0 (1·5, 3·0)
Duration receiving dimethyl fumarate, years; median (quartiles)	2·2 (1·1, 3·2)	1·6 (0·9, 2·3)
**MRI**		
Within-patient number of T2 lesions; median (quartiles)	63 (51, 111)	43 (19·5, 66)
Within-patient size of T2 lesions, mm^2; median (quartiles)	90·6 (67·7, 146·0)	83·96 (55·4, 119·3)
Within-group total number of contrast-enhancing lesions at baseline	3	0
Within-patient lesional MTR, pu; mean (SD)	41·83 (2·03)	41·73 (2·08)
Within-patient brain parenchymal fraction; mean (SD)	0·74 (0·02)	0·75 (0·01)
**VEP**		
Total number of VEP recordings with sufficient quality for inclusion; number of eyes (number of patients)	42 (22)	44 (23)
Participants with history of ON (number of eyes)	11 (12)	14 (20)
Time since ON, years; median (quartiles)	12·2 (4·4, 15·5)	3·3 (1·6, 8·6)
VEP P100 latency, ms; mean (SD)	126·2 (18·6)	119·3 (18·1)

Table 1. Comparison of baseline variables between the two trial arms for all participants in the intention-to-treat analyses. One further participant randomised to bexarotene (who withdrew before month 2) and two further participants randomised to placebo (withdrawn before commencing the IMP) had no follow-up MRI or VEP so could not be included in the intention to treat analyses. EDSS: expanded disability status scale; MTR: magnetization transfer ratio; ON: optic neuritis; VEP: visual-evoked potential.

Table 2. Adverse events in the safety sample

	Bexarotene (n=26)	Placebo (n=24)
**All adverse events**		
Number of adverse events (mean per person)	159 (6·12)	39 (1·63)
Number of participants with ≥1 adverse event (%)	26 (100%)	17 (71%)
Number of participants who discontinued study drug because of adverse event (%)	5 (19%)	2 (8%)
**Serious adverse events**		
Hospitalisation	0	1 (4%) *
**Expected Adverse Effects**		
**Metabolic and nutrition disorders**		
Hypertriglyceridaemia	24 (92%)	0
Secondary (central) hypothyroidism	26 (100%)	0
**Blood and lymphatic system disorders**		
Neutropenia	10 (38%)	0
Lymphopenia	1 (4%)	1 (4%)
**Nervous system disorders**		
Headache	14 (54%)	8 (33%)
**Skin and subcutaneous tissue disorders**		
Rash	13 (50%)	1 (4%)
Pruritis	7 (27%)	0
**Emergent Adverse Effects**		
**Nervous system disorders**		
MS Relapse	1 (4%)	0
MS Pseudorelapse	1 (4%)	4 (17%)
Lhermitte's sign	1 (4%)	0
Cerebellar infarction	1 (4%)	0
Neuropathic pain	1 (4%)	1 (4%)
Muscle spasticity aggravated	1 (4%)	0
Dizziness	1 (4%)	0
Low mood	1 (4%)	0
Memory disturbance	0	1 (4%)
**Skin and subcutaneous tissue disorders**		
Skin desquamation	5 (19%)	0
Dry skin	4 (15%)	0
Acne	1 (4%)	0
Alopecia	1 (4%)	0
Facial flushing	0	2 (8%)
Dry eyes	1 (4%)	0
**Infections and infestations**		
Upper respiratory tract infection	2 (8%)	1 (4%)
Lower respiratory tract infection	1 (4%)	0
Urinary tract infection	2 (8%)	1 (4%)
Shingles	0	1 (4%)

Ear infection	1 (4%)	0
Coryzal symptoms	3 (12%)	4 (17%)
Sinusitis	0	1 (4%)
**Gastrointestinal and hepatobiliary disorders**		
Nausea	5 (19%)	0
Diarrhoea	4 (15%)	4 (17%)
Constipation	2 (8%)	0
Epigastric pain	1 (4%)	0
Dry lips	2 (8%)	0
Ulceration of mouth	2 (8%)	0
Cholecystitis	0	1 (4%)
**Respiratory, thoracic and mediastinal disorders**		
Cough	1 (4%)	1 (4%)
Shortness of breath	0	1 (4%)
Sore throat	1 (4%)	1 (4%)
**Musculoskeletal and connective tissue disorders**		
Stiffness joints	1 (4%)	1 (4%)
Myalgia	1 (4%)	0
**Renal and urinary disorders**		
Nocturia	2 (8%)	0
Urinary frequency	2 (8%)	0
**Vascular disorders**		
Epistaxis	1 (4%)	0
**General disorders**		
Fatigue	6 (23%)	4 (17%)
**Investigations**		
Transaminitis	3 (12%)	0
Weight loss	1 (4%)	0

Table 2. Adverse events in each of the two trial arms for participants who received at least one IMP dose. Unless otherwise stated, values are numbers of participants (%) with at least one event of the stated type. *One patient, on placebo, was hospitalised overnight for treatment of cholecystitis. Expected adverse effects of bexarotene, idenitified from the Summary of Product Characteristics was listed in the trial protocol.

Table 3. Trial MRI outcomes in the intention to treat sample

	Bexarotene		Placebo		Bexarotene-placebo change	
**Subgroup of lesions**	**Patient number**	**Unadjusted mean (SD) change in lesional MTR (pu)**	**Patient number**	**Unadjusted mean (SD) change in lesional MTR (pu)**	**Adjusted bexarotene-placebo difference (95% CI)**	**p-value**
**Primary Efficacy Endpoint (Patient-level)**						
Patient submedian lesion mean**	25	0·25 (0·98)	24	0·09 (0·84)	0·16 (-0·39, 0·71)	0·554
**Pre-specified Exploratory MRI Analyses (Lesion-level)**						
	**Lesion number**	**Unadjusted mean (SD) change in lesional MTR (pu)**	**Lesion number**	**Unadjusted mean (SD) change in lesional MTR (pu)**	**Adjusted bexarotene-placebo difference (95% CI)**	**p-value**
Submedian lesions (defined by cohort-level median)	923	0·35 (2·09)	662	-0·07 (1·68)	0·30 (-0·18, 0·78)	0·223
Supramedian lesions (defined by cohort-level median)	1023	-0·31 (1·74)	562	-0·18 (1·51)	-0·04 (-0·52, 0·43)	0·854
Interaction test comparing treatment group differences between submedian and supramedian lesions	··	··	··	··	··	0·007
Periventricular lesions	205	-0·31 (1·70)	151	-0·18 (1·33)	-0·02 (-0·58, 0·55)	0·953
Deep WM lesions	593	-0·03 (1·72)	356	0·01 (1·39)	-0·06 (-0·56, 0·44)	0·810
Juxtacortical lesions	82	0·09 (1·71)	53	-0·16 (2·15)	0·29 (-0·44, 1·01)	0·441
Leucocortical lesions	650	0 (2·08)	393	-0·02 (1·62)	-0·04 (-0·54, 0·46)	0·867
CGM lesions	46	0·69 (2·58)	39	-0·42 (3·20)	1·00 (0·12, 1·75)	0·023
DGM lesions	7	0·49 (2·81)	9	-1·41 (1·25)	1·93 (0·28, 3·59)	0·027
Mixed DGM and WM lesions	217	0·10 (1·80)	158	-0·24 (1·43)	0·41 (-0·15, 0·97)	0·160
Brainstem lesions	64	0·24 (2·62)	24	-1·21 (1·59)	1·75 (0·86, 2·63)	0·0003
Cerebellar lesions	82	0·04 (2·28)	41	-0·31 (1·54)	-0·03 (-0·79, 0·74)	0·947
Interaction test comparing treatment group differences between lesion locations	··	··	··	··	··	<0·0001
**Prespecified Exploratory Whole-brain MRI Analyses (Patient level)**						
	**Patient number**	**Unadjusted mean (SD) change**	**Patient number**	**Unadjusted mean (SD) change**	**Adjusted bexarotene-placebo difference (95% CI)**	**p-value**

Mean change in lesional MTR (all lesions)	25	0·03 (2·03)	24	-0·11 (2·08)	0·13 (-0·35, 0·62)	0·584
BPF	25	-0·001 (0·003)	24	-0·001 (0·003)	0·0001 (-0·002, 0·002)	0·950
NAWM MTR, pu	25	0·11 (0·64)	24	-0·10 (0·85)	0·18 (-0·26, 0·62)	0·423
GM MTR, pu	25	-0·01 (0·60)	24	-0·09 (0·61)	0·06 (-0·29, 0·41)	0·730
**Prespecified Exploratory Electrophysiological Outcomes**						
	**Number of eyes (patients)**	**Unadjusted latency, ms; mean (SD) change**	**Number of eyes (patients)**	**Unadjusted latency, ms; mean (SD) change**	**Adjusted difference (95% CI)**	**p-value**
All eyes	42 (22)	-2·00 (6·20)	44 (23)	0·70 (4·71)	-2·85 (-5·75, 0·05)	0·054
Eyes with P100 ≤118ms at baseline	13 (7)	1·27 (2·75)	22 (13)	1·01 (5·35)	-0·24 (-4·64, 4·16)	0·916
Eyes with P100 >118ms at baseline	29 (16)	-3·46 (6·78)	22 (12)	0·40 (4·08)	-4·06 (-7·68, -0·44)	0·028
Eyes with P100 >118ms at baseline and no ON in previous 5 years	26 (15)	-3·87 (6·97)	17 (11)	0·08 (4·12)	-4·75 (-8·80, -0·71)	0·032

Table 3. Trial MRI and visual evoked potential (VEP) outcomes for intention to treat analysis. p values and CIs are for the adjusted (for baseline value and prespecified covariates) bexarotene – placebo differences. For the VEP outcomes, one participant in the bexarotene group, and two in the placebo group, contributed one eye to each of the ≤118 ms and >118 ms subgroups. As described in the main text, we prospectively substituted an analysis of BPF for the measure of T1 volume. BPF: brain parenchymal fraction; CGM: cortical grey matter; DGM: deep grey matter; MTR: magnetization transfer ratio; ON: optic neuritis; pu: percentage units; NAWM: normal-appearing white matter; WM: white matter.
